# Successful catheter ablation for sustained ventricular tachycardia in right ventricular infarction after surgery for giant right coronary artery aneurysm: a case report

**DOI:** 10.1093/ehjcr/ytaf097

**Published:** 2025-02-25

**Authors:** Ken Watanabe, Akio Fukui, Tomonori Aono, Satoshi Aita, Motoyuki Matsui

**Affiliations:** Department of Cardiovascular Medicine, Yamagata Prefectural Central Hospital, Yamagata, Japan; Department of Cardiovascular Medicine, Yamagata Prefectural Central Hospital, Yamagata, Japan; Department of Cardiovascular Medicine, Yamagata Prefectural Central Hospital, Yamagata, Japan; Department of Cardiovascular Medicine, Yamagata Prefectural Central Hospital, Yamagata, Japan; Department of Cardiovascular Medicine, Yamagata Prefectural Central Hospital, Yamagata, Japan

**Keywords:** Right ventricular infarction, Giant right coronary artery aneurysm, Ventricular tachycardia, Catheter ablation, Case report

## Abstract

**Background:**

Post-infarction scar-related ventricular tachycardia (VT) originating from the right ventricular (RV) free wall in patients with RV infarction is rare.

**Case summary:**

A 75-year-old Asian male, with a history of RV infarction after surgery for a giant right coronary artery aneurysm, presented with sustained VT with left bundle branch block and inferior axis morphology. The activation mapping during the VT revealed a focal origin initially propagated from the anterior attachment of the RV wall, and mid-diastolic potentials (MDPs) were detected within the RV free wall close to the anterior attachment. At the MDPs recording site, entrainment pacing showed concealed entrainment. The stimulus-QRS interval was equal to the local electrogram-QRS interval. Successful termination of clinical VT was achieved through radiofrequency delivery targeting the MDPs during the VT. Voltage mapping during sinus rhythm (SR) demonstrated a scar area at the RV free wall. Delayed potentials (DPs) during SR were obtained at the zone of MDPs recorded. Additional ablation was performed to eliminate the DPs, resulting in no further inducibility of VT with programmed stimulation.

**Discussion:**

The RV free wall infarction can lead to scar-related arrhythmogenesis. Detailed electrophysiological and functional mapping of such scar areas proved effective in terminating VT.

**Learning points**

- Post-infarction scar-related monomorphic ventricular tachycardia is usually related to left ventricular myocardial infarction, whereas right ventricle can exhibit arrhythmogenicity in patients with right ventricular free wall infarction.
- Electroanatomical mapping is a valuable tool for identifying arrhythmogenic substrates and tachycardia circuits in right ventricular tachycardia.

## Introduction

Sustained ventricular tachycardia (VT) is associated with sudden death and poor clinical outcomes and requires therapeutic intervention.^1^ Myocardial infarction leads to fibrosis and formation of scar tissue, which can serve as a substrate for re-entrant VT.^2^ Post-infarction scar-related monomorphic VT usually occurs after left ventricular myocardial infarction, but it is uncommon for right ventricular (RV) myocardial infarction.^3^ We present an interesting case of a patient with VT originating from the RV free wall after RV infarction following surgery for the giant right coronary artery (RCA) aneurysm.

## Summary figure

**Figure:**
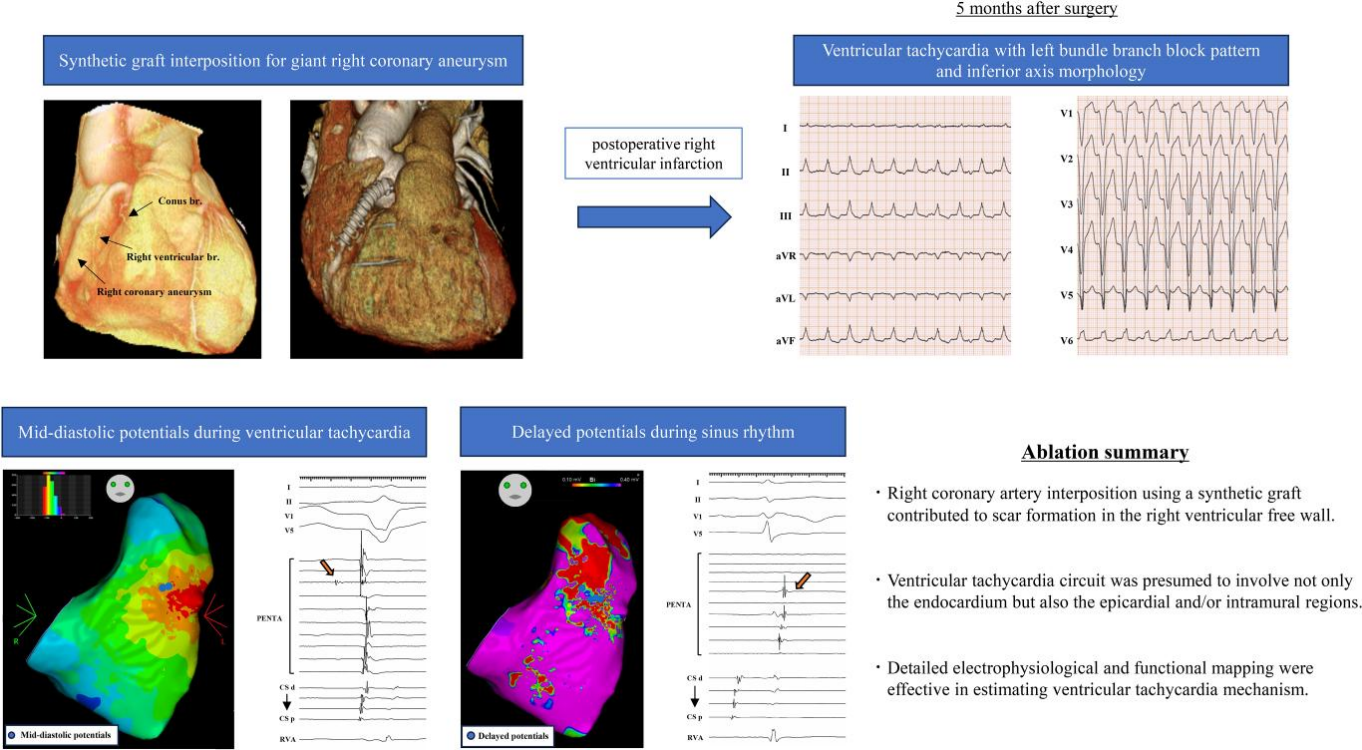


## Case presentation

**Figure 1:**
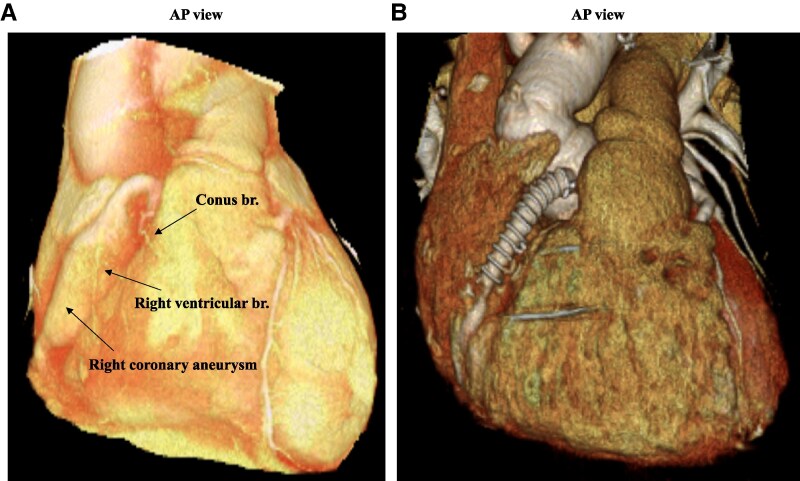
(*A*) Three-dimensional computed tomography shows the giant right coronary artery aneurysm which gives off the conus branch and right ventricular branch. (*B*) Three-dimensional computed tomography shows the right coronary artery synthetic graft interposition for giant right coronary artery aneurysm. AP, anterior-posterior.

A 75-year-old Asian male had acute aortic dissection (Stanford B, DeBakey IIIb) treated with conservative strategy 13 years prior to admission. He had no history of connective tissue diseases or a family history of aortic disease. During the follow-up with contrast-enhanced computed tomography, enlargement of the dissected descending aortic aneurysm and aortic root with severe aortic regurgitation and dilation of the RCA (lesion length, 30 mm) were observed (*Figure 1A*). Elective Bentall surgery, total arch replacement, and RCA synthetic graft interposition for giant coronary aneurysm were performed 5 months before admission (*Figure 1B*). After coronary artery bypass grafting (CABG), he experienced RV infarction and subsequent right heart failure. His arterial blood pressure was 90/50 mmHg under multiple administrations of catecholamines and required large volumes of intravenous fluids and blood transfusions during the acute phase. Finally, he was discharged 61 days after cardiac rehabilitation.

**Figure 2:**
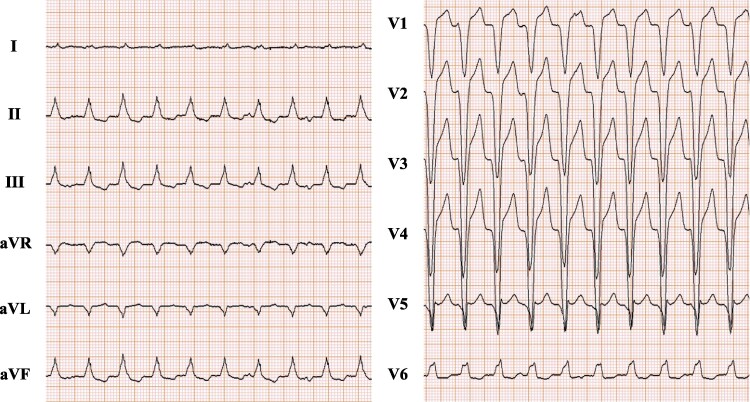
Twelve-lead electrocardiogram during ventricular tachycardia at 132 b.p.m with left bundle branch block morphology and inferior axis deviation. Electrocardiogram was recorded at a paper speed of 25 mm/s with an amplitude of 10 mm/mV.

He presented with palpitation and was admitted to our hospital for sustained VT. His 12-lead electrocardiogram during VT showed at 132 b.p.m with left bundle branch block (LBBB) pattern and inferior axis morphology (*Figure 2*). He maintained an oxygen saturation of 97% on room air and exhibited clear breath sounds without heart murmur. Laboratory tests confirmed an elevated creatinine of 1.16 mg/dL and brain natriuretic peptide of 736 pg/mL. Transthoracic echocardiography showed akinesia of the RV free wall, enlargement of right ventricle, mild tricuspid regurgitation, and mild pericardial effusion.

**Figure 3:**
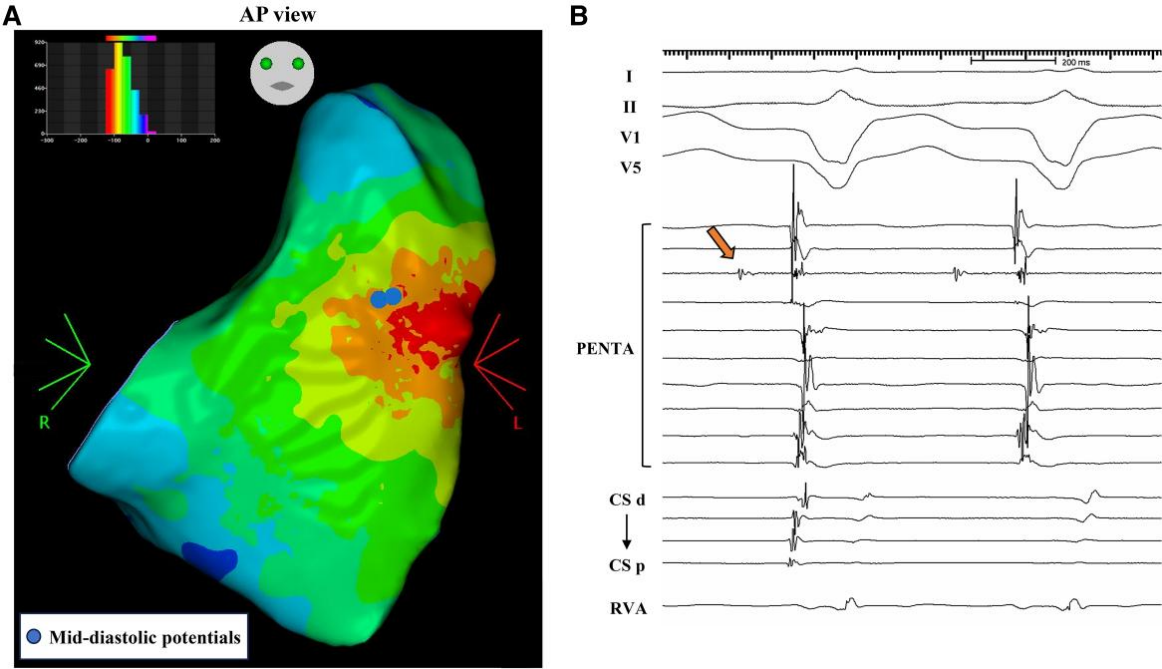
(*A*) Activation mapping during VT shows the focal propagation from the anterior attachment of the right ventricular wall. Mid-diastolic potentials are obtained in close to the anterior attachment (tag). (*B*) Cardiac tracing of mid-diastolic potentials during the ventricular tachycardia is shown (arrow). MDPs, mid-diastolic potentials; VT, ventricular tachycardia.

**Figure 4:**
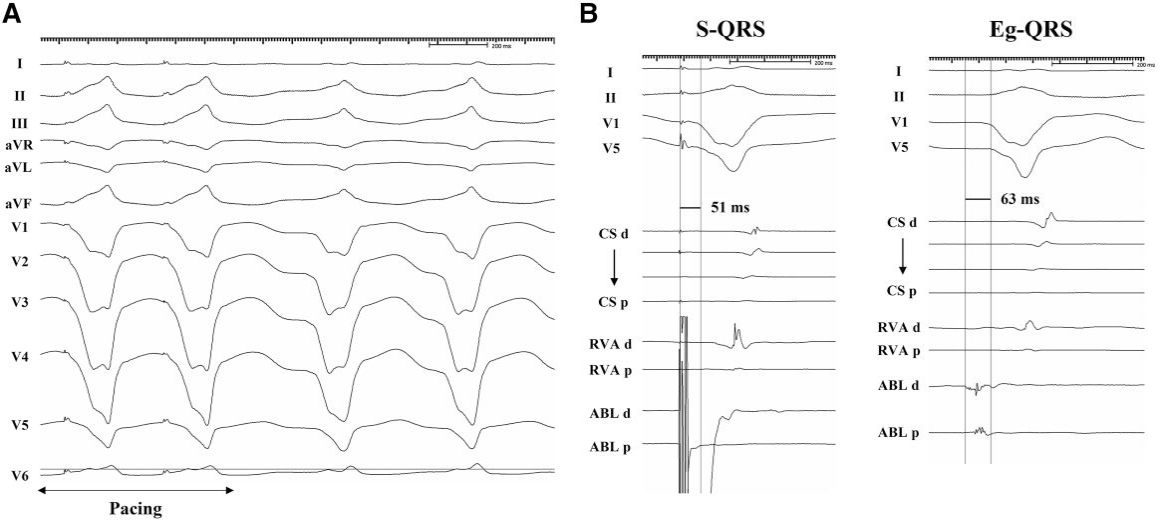
(*A*) Entrainment at the mid-diastolic potentials recording site shows concealed fusion. (*B*) The stimulus-QRS interval is nearly equal to the local electrogram-QRS interval. MDPs, mid-diastolic potentials.

**Figure 5:**
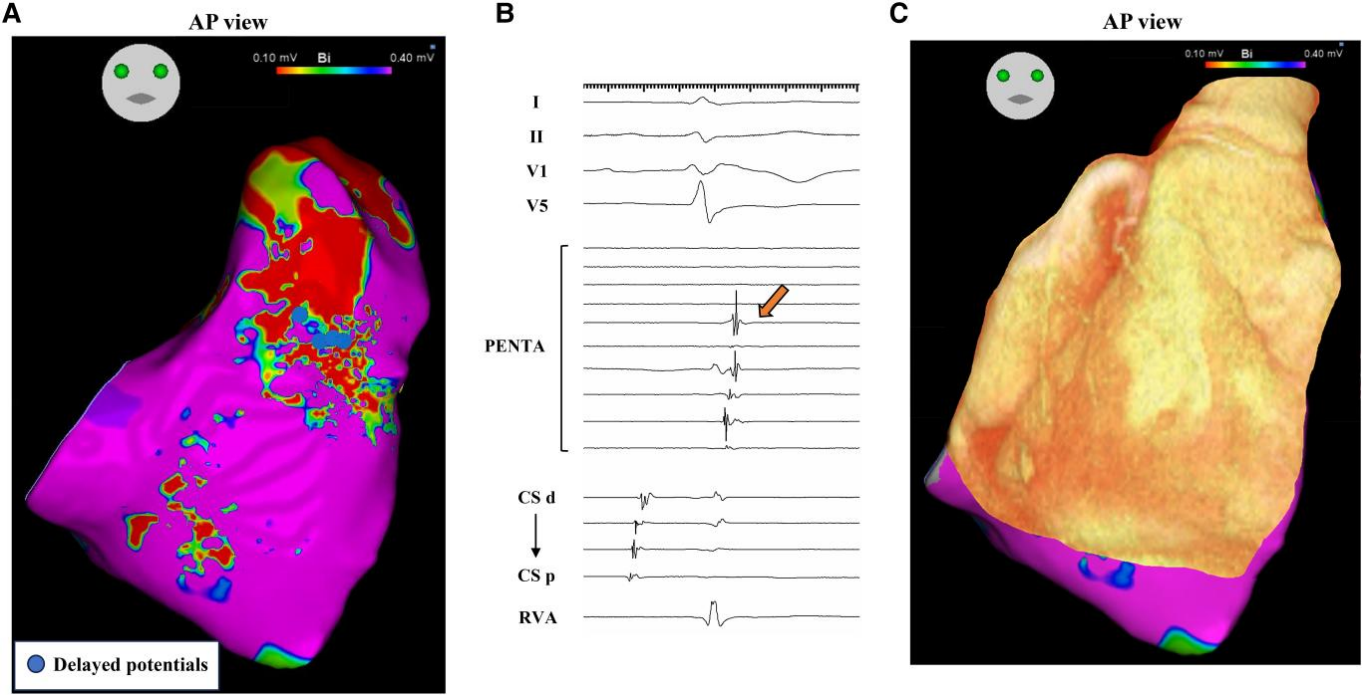
(*A*) Voltage mapping during SR. Delayed potentials are located within the dense scar area in the right ventricular free wall (tag). (*B*) Cardiac tracing of delayed potentials during the sinus rhythm is shown (arrow). (*C*) Three-dimensional computed tomography merged with the voltage mapping during SR. AP, anterior-posterior; DPs, delayed potentials; SR, sinus rhythm.

Antiarrhythmic drugs (amiodarone and landiolol) were administered to stabilize the VT. Despite pharmacological treatment, the VT persisted and necessitated use of external defibrillation. Unfortunately, the VT recurred repeatedly, resulting in a state of electrical storm. A catheter ablation procedure was planned for the electrical VT storm. Electrophysiological studies and radiofrequency catheter ablation were performed using a 3D mapping system (CARTO 3 Version 7, Biosense Webster, Inc., Diamond Bar, CA, USA). The clinical VT with LBBB and inferior axis QRS morphology and a cycle length of 510 ms persisted at the start of the ablation procedure. Activation mapping during VT revealed a focal activation pattern, with the earliest activation occurring at the anterior attachment of the RV wall (*Figure 3A*), and the mid-diastolic potentials (MDPs) were detected within the RV free wall close to the anterior attachment (*Figure 3B*). At the MDPs recording site, entrainment pacing showed concealed entrainment (*Figure 4A*). The stimulus-QRS (S-QRS) interval was matched to the local electrogram-QRS (Eg-QRS) interval (51 ms, 10% of the VT cycle length), indicating that the site was located near the VT exit (*Figure 4B*). Based on these observations, the RV free wall was found to be a critical isthmus of the VT and the RV anterior attachment was the exit point of VT re-entry circuit. Successfully, irrigated radiofrequency ablation (30 W, contact force 10–15 g) targeting MDPs in the RV free wall terminated the VT immediately, while the radiofrequency ablation of the exit point was insufficient to terminate the VT. Voltage mapping during sinus rhythm (SR) demonstrated a dense scar area (<0.1 mV) at the RV free wall. Delayed potentials (DPs) were obtained during SR in the scar area, where MDPs were located (*Figure 5A* and *B*). Pacing from near the earliest activation site exhibited a good QRS match (PASO match score of 96) to the targeted VT morphology with stimulus-to-QRS latency. Additional radiofrequency ablation was performed to eliminate the DPs, resulting in no further inducibility of VT with programmed ventricular stimulation. The patient was discharged without any acute or periprocedural complications. During the 5-month follow-up, the patient has been free from VT recurrence.

## Discussion

The most common cause of sustained monomorphic VT is myocardial infarction-related scar tissue,^4^ which can be life-threatening and requires emergency medical treatment. Most cases of post-infarction VT are associated with the left ventricle; however, they are rarely observed in the right ventricle. One potential explanation for their rarity is that the RV muscle is thinner and therefore has less volume of the infarcted muscle compared to the left ventricular muscle, which might contribute to the right ventricle not forming a re-entrant VT circuit.^3^ In addition, it was reported that the right ventricle relatively resistant to ischaemia and can often recover contractility after ischaemic reperfusion injury, but its haemodynamic and electrical instability is more pronounced during the acute phase of RV infarction.^5^

The present patient had suffered from RV infarction after CABG for giant coronary artery aneurysm using synthetic graft material. As shown in *Figures 2A* and *B* and *5C*, the conus branch and RV branch were occluded by the synthetic graft interposition, which may have contributed to scar formation in the RV free wall, serving as a substrate for scar-related arrhythmogenesis. Similar to left ventricular originated VT, the pathophysiology of RV originated VT in old myocardial infarction involves re-entry around a scar, with slow conduction areas forming the VT isthmus.^6^ Detailed three-dimensional electroanatomic mapping has been reported to be helpful in describing post-infarction scar-related re-entrant VT circuits.^7^ In this case, activation mapping during VT and substrate mapping during SR contributed to estimating the VT mechanism. Although the VT demonstrated a focal pattern with the earliest activation occurring at the anterior attachment of the RV wall and the activation time distribution was incomplete, the phenomenon of entrainment of the VT suggested a re-entry mechanism. The MDPs were recorded next to the earliest activation site, where radiofrequency delivery resulted in immediate termination of the VT. This supports the assessment that the MDPs recorded at this site reflected an essential part of the re-entrant pathway of the VT. Additionally, the association between the S-QRS and the Eg-QRS indicated that this site was the VT isthmus and exit portion. Based on these electrophysiological findings, the VT circuit was presumed to involve not only the endocardium but also the epicardial and/or intramural regions. Although epicardial and/or intramural involvement are more common in non-ischaemic cardiomyopathy-related VT,^8^ it has been reported that mid-myocardial or epicardial layers could be origins for post-infarction scar-related VT.^9^ In this case, the VT was successfully treated with endocardial ablation at 30 W. Increasing radiofrequency power or extending energy delivery time should be considered if the initial treatment was insufficient.^8^ A percutaneous epicardial approach is often indicated for patients who previously failed endocardial ablation. An epicardial approach may be required for treating VT in patients with non-ischaemic cardiomyopathy and, occasionally, in those with post-myocardial infarction.^10^ Due to its high potential for complications, careful consideration of treatment selection is necessary to prevent side effects, especially in less experienced centres.^11^

## Conclusion

A post-infarcted scar in the RV free wall, as well as the left ventricle, can lead to scar-related arrhythmogenesis. In this case, RCA interposition using a synthetic graft contributed to scar formation in the RV free wall. Detailed electrophysiological and functional mapping during both SR and VT effectively eliminated the VT.

## Data Availability

The data underlying this article will be shared on reasonable request to the corresponding author.

## Lead author biography

Dr Ken Watanabe earned his MD and PhD from Yamagata University School of Medicine and is currently a physician at Yamagata Prefectural Central Hospital. He has a strong interest in catheter ablation for cardiac arrhythmias.

## Notes

**Consent:** The authors confirm that written informed consent for submission and publication of this clinical case report has been obtained from the patient in line with the COPE guidelines.

**Funding:** None declared.
